# Childhood intra- and extra-familial maltreatment and later-life trajectories of depressive symptoms: evidence from China

**DOI:** 10.1186/s12877-024-05169-w

**Published:** 2024-07-12

**Authors:** Tingshuai Ge, Yixiao Liu, Qing Han, Xinfeng Cheng, Quanbao Jiang

**Affiliations:** 1 School of Public Policy and Administration, Xi’an Jiaotong University, Xi’an, China; 2 School of Public Policy and Administration, Center for Public Economy & Public Policy, Chongqing University, Chongqing, China; 3 School of Economics and Management, Xi’an Technological University, Xi’an, China

**Keywords:** Childhood maltreatment, Depressive symptoms, Trajectories, Middle-aged and older adults, China

## Abstract

**Background:**

Both late-life depression and childhood maltreatment have become major global public health issues, given their prevalence and social-economic and health consequences. However, previous studies have solely focused on the relationship of childhood maltreatment to average levels of depressive symptoms. The current study addresses this gap of knowledge by simultaneously examining the impacts of childhood intra- and extra-familial maltreatment on age trajectories of depressive symptoms in later life in the Chinese context.

**Methods:**

Hierarchical linear models were applied to data from the China Health and Retirement Longitudinal Study (2011–2018, *N* = 12,669 individuals aged 45 to 80, comprising *N* = 43,348 person-years). Depressive symptoms were measured by the CES-D-10 scale. Childhood intra-familial maltreatments were measured by physical abuse and emotional neglect, while extra-familial maltreatment was measured by peer bullying. All analyses were conducted separately by gender in Stata 16.

**Results:**

Childhood extrafamilial peer bullying (β = 1.628, *p* < 0.001), and intrafamilial physical abuse (β = 0.746, *p* < 0.001) and emotional neglect (β = 0.880, *p* < 0.001) were associated with higher later-life depressive symptoms levels in the whole sample. Peer bullying differences in depressive symptoms widened with age for both men and women. Physical abuse differences in depressive symptoms remained stable over the life course among men but increased among women. Emotional neglect differences in depressive symptoms decreased with age among men, while it increased first and then decreased among women.

**Conclusions:**

Findings in this study suggest that childhood maltreatment is not only associated with later-life poorer mental health but contributes to increasing inequalities in mental health as people age, especially among peer-bullying victims and women.

**Supplementary Information:**

The online version contains supplementary material available at 10.1186/s12877-024-05169-w.

## Background

Depression in later life has emerged as a significant global public health concern. As estimated, 5.7% of people aged 60 and above worldwide suffered from depression [1]. The prevalence of depression among older adults is 14.6% in the United States, 17.6% in the United Kingdom, and 19.8% in Japan [2, 3]. In China, many meta-analyses indicate a depression prevalence exceeding 20% in older adults, which is higher than that in developed countries [4, 5]. Documented evidence have suggested that late-life depression can lead to poorer quality of life [6], increased medical expenditures [7], and elevated mortality rates [8], rendering it the world’s second leading global disease burden [9].

Childhood maltreatment has also been recognized as a major public issue due to its substantial prevalence and consequential socio-economic and health ramifications. Child maltreatment encompasses a range of forms of abuse, neglect, and exploitation perpetrated by people such as parents or peers, resulting in tangible or potential harm to children’s well-being, survival, development, or dignity [10]. Among these, intra-familial physical abuse, emotional neglect, and extra-familial peer bullying stand out as the most prevalent forms of childhood maltreatment [11, 12]. Globally, the prevalence of physical abuse is 17.7%, and emotional neglect is 18.4% [13]. The incidence of peer bullying ranges from 13 to 30% in developed nations such as the United States and Europe [14, 15]. In less developed regions such as China, the prevalence is even higher: the occurrence of physical abuse is reported at 26.6%, neglect at 26.0%, and peer bullying at 25-55% [16–18], with indications of ongoing escalation [19]. A plethora of research has substantiated that childhood maltreatment not only exerts enduring adverse effects on people’s physical and mental health but brings serious economic losses to society [10, 17].

Levels of depression in later life has been shown to be associated with childhood maltreatment. Numerous studies from Western developed countries have demonstrated that the adverse impact of childhood maltreatment on depressive symptoms levels will last into later life [20–23]. For instance, a study in the United Kingdom revealed that exposure to peer bullying at ages 7–11 was associated with a higher risk of depression at age 45 and higher levels of psychological distress at age 50 [21]. Research from the Netherlands found associations between childhood physical abuse and emotional neglect with depression onset in early (< 40 years), middle (40–60 years), and late (≥ 60 years) adulthood [20]. Similar conclusions have been drawn from studies in China [12, 24, 25] as well as multiple meta-analyses [26, 27]. The relationship between childhood maltreatment and late-life depressive symptoms also displays gender differences. Men are more likely to experience physical abuse and peer bullying, while women are more susceptible to emotional neglect [28] and demonstrate a greater vulnerability to the negative impacts of childhood maltreatment [19, 29].

However, prior research can be improved in the following aspects: Firstly, few studies have simultaneously analyzed the effects of intra- and extra-familial childhood maltreatment. Both parents and peers play a vital role in children’s socialization processes: parents serve as crucial sources of protection, support, and security for children, while children spend most of their time outside the family socializing and interacting with peers [25]. This difference could give rise to significant and distinct impacts on mental health stemming from occurrences of child maltreatment both within and outside the familial environment. Furthermore, as previous research suggested, children subjected to peer bullying frequently experience other forms of maltreatment at home, followed by further maltreatment from peers or adults, creating a cycle of violence and abuse [30]. Nonetheless, much of the existing research has primarily focused on examining the impact of childhood maltreatment within specific contexts, such as the home, school, or other settings [20, 25], without simultaneously contrasting the differences in effects associated with childhood intra- and extra-familial maltreatment.

Secondly, limited understanding exists regarding how a history of childhood maltreatment shapes trajectories of depressive symptoms in later life. The cumulative advantage/disadvantage hypothesis (CAD) posited that initial advantages or disadvantages accumulate over time, resulting in accumulating inequalities in access to resources and exposure to risks [31]. Applied to the relationship between childhood maltreatment and mental health, the CAD predicts an intracohort increase in mental health disparities between people with and without childhood maltreatment experiences. However, most previous studies relied on cross-sectional data and solely examined the impact on the average level of depressive symptoms [23, 32, 33]. It is imperative to investigate whether the childhood maltreatment differences in later-life depressive symptoms levels widen or diminish with age, which could aid in identifying maltreatment types that consistently exert adverse effects and enable targeted interventions.

Finally, it is essential to examine the influence of childhood maltreatment on the age trajectories of late-life depressive symptoms in the Chinese context, where the socio-economic and cultural background significantly diverges from Western nations. For instance, before 1970, China’s total fertility rate was about six births per woman [34], leading children susceptible to parental neglect. Before the 1990s in China, societal norms sanctioned parental corporal punishment of children as a means of discipline practice [24]. Under the long-term influence of these cultural norms and social background, Chinese children tend to rationalize parental abusive behaviors and unconsciously defend their parents [35], which may potentially impact individuals’ psychological responses to childhood maltreatment. Furthermore, the current older population in China has experienced significant societal changes and historical events over their lifetimes, such as wars (before 1949), the Great Famine (1959–1961), and the Cultural Revolution (1966–1976). These experiences might have honed the stress-coping capacities, amplifying or mitigating the effects of childhood maltreatment on late-life depressive symptoms. Thus, it is critical to explore the effects of childhood maltreatment on mental health trajectories in the Chinese context and learn how they may differ from Western countries.

At present, only limited studies have analyzed the impact of childhood maltreatment on late-life health trajectories in China, but these studies were based on physical health measures such as functional limitations [36] and multimorbidity [37, 38]. In terms of these limitations, we used a five-wave (2011–2018) data from the China Health and Retirement Longitudinal Study (CHARLS) to simultaneously analyze the impact of childhood intra-familial (physical abuse, emotional neglect) and extra-familial maltreatment (peer bullying) on later-life depressive symptoms. Two questions were addressed in this study: (1) Does childhood intra- and extra-familial maltreatment affect average levels of depressive symptoms in later life? (2) How do childhood intra- and extra-familial maltreatment shape the age trajectories of depressive symptoms in later life? Considering the significant differences have been reported between men and women in terms of the risk of exposure to childhood maltreatment, coping strategies in response to maltreatment, as well as the average levels and trajectories of depressive symptoms [28, 39, 40], we conducted our analysis separately for men and women.

## Methods

### Data

Data used in this study was from the CHARLS, a nationally representative survey of Chinese adults aged 45 years and above. The baseline survey was conducted in 2011, with four follow-up surveys were carried out in 2013, 2014, 2015, and 2018, respectively. In the 2014 survey, CHARLS conducted a Life History Survey covering the history of family. All information were collected via face-to-face interviews, with a follow-up rate of more than 80%.

We used five-wave (2011–2018) data of CHALRS to examine the impacts of childhood maltreatment on later-life depressive symptoms trajectories. We only used information from individuals who completed the 2014 Life History Survey, with 14,422 individuals and 53,825 observations. After excluding people (1) aged under 45 or above 80 (to retain enough data for estimating the average levels of depressive symptoms at each specific age–cohort–gender cell, we constrained the sample to participants aged up to 80 at the time of the interviews; individuals = 650, observations = 3,436); (2) had missing values in childhood maltreatment and gender (individuals = 939, observations = 2,697); (3) had two or more missing items of the CES-D-10 scale (individuals = 164, observations = 4,344), our final analytic sample includes 12,669 participants, 43,348 observations.

### Measures

#### Depressive symptoms

Depressive symptoms (CESD) were measured by a 10-item short form of the Center for Epidemiologic Studies Depression Scale (CES-D-10). Participants were asked about the frequency of depressive symptoms during the last week, with four options ranging from “none of the time” (0) to “most of the time” (3) available for each CES-D-10 item. Referring to previous studies [41, 42], we allowed one missing item in the CES-D-10 scale and then substituted the mean for the missing item, which does not change the scale integrity. CESD score (0–30) was obtained by calculating the total score of ten items, with a higher score indicating higher levels of depressive symptoms (Cronbach’s alpha ≥ 0.75 in 2011–2018).

#### Childhood maltreatment

We focused on three common types of childhood maltreatment in this study: peer bullying (extra-familial maltreatment), parental physical abuse and emotional neglect (intra-familial maltreatment). Different types of childhood maltreatment experienced up to the age of 17 were assessed based on self-reports in CHARLS.

Peer bullying was measured by the question: “When you were a child, how often were you picked on by kids in the school/neighborhood”, with four options ranging from 1 (often) to 4 (never) available. Peer bullying was treated as “yes” if the answer was “often or sometimes”, otherwise, treated as “no”.

Physical abuse was measured by the question: “When you were growing up, did your female/male guardian ever hit you?”, with four responses ranging from 1 (often) to 4 (never) provided. Physical abuse was treated as “yes” if the answer was “often or sometimes”, otherwise, treated as “no”.

Emotional neglect was measured by six questions: “How much love and affection did your female guardian give you while you were growing up?”, “How much effort did your female guardian put into watching over you?”, “How strict was your female/male guardian with her/his rules for you?”, “Did your female/male guardian treat your siblings better than you when you were growing up?”. The responses range from 1 (often) to 4 (never) for each question. The total score (6–24) was calculated, with a higher score indicating higher levels of neglect. Emotional neglect was treated as “yes” if the total score was at least one standard deviation above the average, otherwise, treated as “no”. This is one of the common cutoff specifications used in previous studies [43–45].

#### Age and cohort

As a time-varying variable, age ranges from 45 to 80. Birth cohort was calculated as a time-constant variable that indicated the year of birth. It was minimum-centered and ranged from 0 to 35, corresponding to a birth cohort period from 1931 to 1966. Previous studies suggested that birth cohort is a proxy for the historical periods to which individuals were exposed, and age trajectories of CESD may vary across cohorts considering different risk factors for mental health that each cohort was exposed to [46, 47]. Thus, we also controlled for cohort in our models to accurately estimate the association between childhood maltreatment and age trajectories of CESD. The parameterizations of age and cohort effects on CESD were determined by referring to previous studies [45, 48]. We finally included linear terms of age, cohort, and the interaction term between age and cohort in the analytic model for both men and women.

#### Gender

Gender was coded as 1 for men and 0 for women. Descriptive statistics of all variables included in our analyses were presented in Table 1.

### Analytic strategy

We used hierarchical linear models (HLM) to estimate the impacts of childhood maltreatment on CESD. HLM estimation enables the trajectories of CESD to have different average levels (random intercepts) and rates of change (random slopes). Thus, they provide information about mean CESD trajectories as well as individual differences in these trajectories.

The following analyses began with a description of the sample. Then we analyzed the impacts of childhood maltreatment on average levels of CESD. We next examined three different types of childhood maltreatment on age trajectories of CESD. Given the gender differences in mental health trajectories [40, 49], separate models were conducted for men and women.

We also conducted many analyses to check the robustness of our results. Firstly, prior research has indicated that children subjected to peer bullying may be vulnerable to parental maltreatment, and both forms of maltreatment collectively contribute to worse mental health later in life [30, 50]. Therefore, we controlled for other types of maltreatment when analyzing the effect of each specific type of childhood maltreatment.

Secondly, previous studies have suggested that people who had childhood maltreatment experiences are more likely to have lower levels of education [12, 21], unfavorable health behaviors [28], and poorer health conditions [36, 37]. Thus, we also examined the role of educational attainment, health behaviors (smoking, drinking, and social participation), and health conditions (self-reported health, Instrumental Activities of Daily Living, and number of chronic diseases) in the association between childhood maltreatment and CESD trajectories. The definition and measurement of these variables were detailed in Table S1.

Thirdly, we investigated the effects of missing values of childhood maltreatment and CESD. For childhood maltreatment, we categorized missing values into a separate group and included them in the models rather than excluding them. Regarding CESD, we employed Multiple Imputation (MI) to fill in the missing values in CESD. MI is a commonly used method for handling missing data, where missing values are imputed multiple times using a statistical model based on the available data [51]. We used variables in M1 of Table 2 to impute missing CESD values, with 20 imputations generated.

Finally, we applied the Inverse Probability Weighting (IPW) to correct the potential bias due to sample attrition. IPW can give higher influence to individuals with a high probability of dropping out and lower influence to those with a lower probability of dropping out [52]. In this study, the variables used to calculate IPWs were CESD, age, three types of childhood maltreatment, and interaction terms between age and childhood maltreatment measured at *t*-1. We calculated the weights separately for men and women. All analyses were conducted in Stata Version 16.

## Results

### Descriptive results

Table 1 provides the sample characteristics. In the whole sample, the average CESD was 8.36, and the prevalence of peer bullying, physical abuse, and emotional neglect were 15.05%, 28.73%, and 12.16%, respectively. Women had a higher level of CESD (9.41 vs. 7.21, *p* < 0.001) but a lower prevalence of peer bullying (13.24% vs. 17.07%, *p* < 0.001) and physical abuse (23.60% vs. 34.46%, *p* < 0.001) than men, while there was no gender difference in the emotional neglect prevalence (12.21% vs. 12.11%, *p* = 0.863).

**Table 1:** Sample characteristics

Variables	Whole sample		Men		Women		*p*-value ^c^
	Mean / %	SD	Mean / %	SD	Mean / %	SD	
*Participant characteristics* ^*a*^							
Gender							
Men	47.20%						
Women	52.80%						
Peer bullying							0.000
No	84.95%		82.93%		86.76%		
Yes	15.05%		17.07%		13.24%		
Physical abuse							0.000
No	71.27%		65.54%		76.40%		
Yes	28.73%		34.46%		23.60%		
Emotional neglect							0.863
No	87.84%		87.89%		87.79%		
Yes	12.16%		12.11%		12.21%		
Birth year	1952.76	8.55	1952.12	8.57	1953.33	8.49	0.000
*Observation characteristics* ^*b*^							
Age	60.69	8.28	61.26	8.27	60.17	8.25	0.000
Survey year	2014.07	2.54	2014.08	2.54	2014.06	2.55	0.388
Number of waves	2.43	1.11	2.43	1.11	2.42	1.11	0.309
CESD	8.36	6.33	7.21	5.74	9.41	6.66	0.000

*Notes*: ^a^ Time-constant variables are summarized at the 2011 baseline. ^b^ Time-varying variables are summarized over all observations. ^c^*Chi-square* test for categorical variables and *t* test for continuous variables were conducted to examine the gender difference in sample characteristics. CESD = Depressive symptoms. SD = Standard Deviation

### Childhood maltreatment and depressive symptoms

Table 2 shows the impact of childhood maltreatment on average levels of CESD. Consistent with previous research, suffering from peer bullying, physical abuse, and emotional neglect during childhood were associated with higher CESD levels in later life for both genders. Of the three types of childhood maltreatment, peer bullying had the greatest adverse impact on mean levels of CESD, followed by emotional neglect and finally physical abuse in both men and women.

**Table 2:** Results of the hierarchical linear model for average CESD levels

Variables	M1: Whole sample		M2: Men		M3: Women	
	β	*p*-value	β	*p*-value	β	*p*-value
Gender	2.393^***^	0.000				
	(0.089)					
Age	0.023	0.143	0.012	0.573	0.036	0.124
	(0.016)		(0.020)		(0.023)	
Cohort	-0.030^*^	0.041	-0.035^+^	0.073	-0.022	0.308
	(0.015)		(0.020)		(0.021)	
Age # Cohort ^a^	0.003^***^	0.000	0.002***	0.001	0.004***	0.000
	(0.001)		(0.001)		(0.001)	
Peer bullying	1.628^***^	0.000	1.500***	0.000	1.766***	0.000
	(0.125)		(0.157)		(0.194)	
Physical abuse	0.746^***^	0.000	0.708***	0.000	0.782***	0.000
	(0.100)		(0.125)		(0.158)	
Emotional neglect	0.880^***^	0.000	0.892***	0.000	0.852***	0.000
	(0.136)		(0.179)		(0.203)	
Constant	5.922^***^	0.000	6.514***	0.000	7.730***	0.000
	(0.451)		(0.599)		(0.663)	
Observations	43,348		20,690		22,658	

*Notes*: ^a^ refers to the interaction between age and cohort. CESD = Depressive symptoms. Standard errors in parentheses underneath the coefficients. ^+^*p* < 0.1, ^*^p < 0.05, ^**^p < 0.01, ^***^p < 0.001

**Fig. 1:**
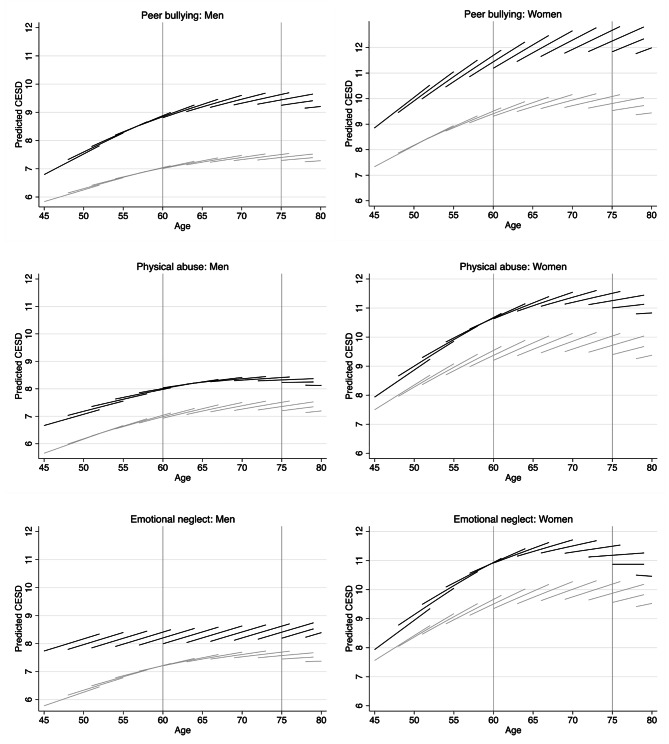
Predicted aging vectors of CESD. *Notes*: Predictions are based on Models 1–6 in Table S2 of the supplemental material. CESD = Depressive symptoms. Black lines = Exposed to childhood maltreatment. Gray lines = Not exposed to childhood maltreatment

We next examined three forms of childhood maltreatment on the age trajectories of CESD in later life. The results from HLM models are shown in Table S2. In the presence of twofold and threefold interaction effects, the coefficients in Table S2 cannot be interpreted straightforwardly. To facilitate the interpretation, we present age-vector graphs (Fig. 1) that show age-related changes in average CESD levels for different birth cohorts of people with and without childhood maltreatment experiences.

Figure 1 shows model-based predictions for change in CESD by childhood maltreatment and gender. CESD trajectories of people exposed to childhood maltreatment are represented by black curves, and those not exposed by the gray curves. The y-axes of the figures are defined for the predicted average values of CESD at fixed values of age, cohort, and childhood maltreatment, with higher values indicating higher CESD. To allow for cohort effects, we fixed the variable for cohort at 12 values of age at initial observation, counting in three-year intervals from the age of 45 (i.e., birth year of 1966) to the age of 80 (i.e., birth year of 1933). As a result, the plots show cohort-specific curves, whereby the length of each curve indicates each cohort’s age at the beginning and at the end of their observation period.

To evaluate age patterns and effect sizes, we compared childhood maltreatment differences at the initial age of 45 with childhood maltreatment differences at the age of 60 and 75, indicated by reference lines in each plot. According to the age classification issued by the World Health Organization in 2015 [53], the ages of 45, 60, and 75 represent the starting points of these three distinct life stages. Upon entering these life stages, individuals may increasingly encounter changes in physical functioning, cognitive function, and other factors that could impact their psychological well-being [54]. Consequently, these age ranges cover an important part of the later life course, during which childhood maltreatment differences in mental health may unfold. To assess variability in the estimates, Table 3 shows the corresponding marginal effects for childhood maltreatment differences along with their confidence intervals for CESD at the initial age of 45 and at the age of 60 and 75 as well as change in childhood maltreatment differences in CESD across these age intervals.

Three primary findings have emerged from the results, as shown in Fig. 1 and Table 3. Firstly, peer bullying differences in CESD escalate with age for both men and women. Participants subjected to peer bullying consistently exhibited significantly higher levels of CESD at ages 45, 60, and 75 compared to those not subjected to such experience. Between ages 45 and 60, disparities in CESD associated with peer bullying increased by 0.840 in men (0.360 in women); by age 75, the disparities elevated by 0.993 from age 45 in men (0.782 in women).

Secondly, physical abuse differences in CESD remained stable across ages in men and increased in women. Among men, participants who suffered from physical abuse demonstrated significantly higher CESD levels at ages 45, 60, and 75 in comparison to those who did not undergo such abuse. The initial physical abuse differences in CESD observed at age 45 persisted consistently over the subsequent three decades (up to age 75). Among women, while there were no significant differences in CESD associated with physical abuse at age 45, significant disparities emerged at ages 60 and 75. Over the thirty-year span from age 45 to 75, the divergence in CESD associated with physical abuse gradually intensified.

Lastly, the disparities in CESD associated with emotional neglect exhibited a declining trend with age in men, while they displayed an initial increase followed by a subsequent decrease in women. For men, participants subjected to emotional neglect displayed significantly higher CESD levels at ages 45 and 60, while there were no significant differences in CESD by age 75. Across the age range from 45 to 75, the emotional neglect differences in CESD gradually diminished. In contrast, there were no significant differences in CESD associated with emotional neglect at age 45 among women. However, significant disparities were evident at ages 60 and 75. From age 45 to 75, the CESD disparities related to emotional neglect initially increased and subsequently decreased.

**Table 3:** Marginal childhood maltreatment differences in CESD at age 45, 60 and 75 by gender

CESD differences	Peer bullying		Physical abuse		Emotional neglect	
	Men	Women	Men	Women	Men	Women
At age 45	0.960^*^	1.518^***^	1.010^**^	0.436	1.956^***^	0.377
	[0.129, 1.791]	[0.598, 2.438]	[0.316, 1.704]	[-0.351, 1.222]	[0.985, 2.927]	[-0.637, 1.390]
At age 60	1.800^***^	1.878^***^	1.107^***^	1.433^***^	0.785^**^	1.571^***^
	[1.325, 2.275]	[1.271, 2.486]	[0.738, 1.476]	[0.964, 1.902]	[0.235, 1.334]	[0.958, 2.183]
At age 75	1.953^***^	2.300^***^	1.032^**^	1.608^***^	0.744	1.313^*^
	[1.013, 2.893]	[0.960, 3.639]	[0.335, 1.728]	[0.630, 2.586]	[-0.324, 1.813]	[0.134, 2.493]
Change from age 45 to 60	0.840	0.360	0.097	0.997^+^	-1.171^+^	1.194^+^
	[-0.221, 1.901]	[-0.877, 1.597]	[-0.767, 0.960]	[-0.019, 2.014]	[-2.409, 0.067]	[-0.117, 2.505]
Change from age 45 to 75	0.993^+^	0.782	0.022	1.172^*^	-1.212^+^	0.936
	[-0.149, 2.134]	[-0.677, 2.240]	[-0.872, 0.915]	[0.032, 2.313]	[-2.520, 0.097]	[-0.494, 2.368]
Observations	20,690	22,658	20,690	22,658	20,690	22,658

*Notes*: CESD = Depressive symptoms. Average marginal differences are shown. Values in brackets are 95% confidence intervals. Estimates are based on Models 1–6 in Table S2 of the supplemental material. CESD differences at ages 45, 60, and 75 are predicted mean differences in CESD between participants with and without childhood maltreatment. Positive values indicate higher CESD levels among participants with and without childhood maltreatment. Change with age is calculated as a difference between the predicted mean differences at age 45 and the predicted mean differences at age 60 or 75. ^+^*p* < 0.1, ^*^p < 0.05, ^**^p < 0.01, ^***^p < 0.001

### Robustness checks

Many additional analyses were conducted to examine the robustness of our results. We first examined the joint effects of childhood intra- and extra-familial maltreatment on age trajectories of CESD. Our conclusion did not change, indicating that different types of childhood maltreatment independently influence the age trajectories of CESD, independent of other adverse childhood experiences (results not presented). Secondly, we examined the role of educational attainment, health behaviors, and health conditions in the association between childhood maltreatment and CESD trajectories. Results indicated that only after controlling for health status did the CESD trajectories change, yet it did not alter our main conclusion (see Figure S1). For example, for the emotional neglect among men in Figure S1, there was a decrease in the average CESD levels across all cohorts among those who experienced emotional neglect after controlling for health status. However, the conclusion that differences in CESD associated with emotional neglect showed a declining trend with age remained unchanged. Finally, we investigated the effects of missing values of childhood maltreatment and CESD, as well as the impact of potential selective bias, and our findings remained unchanged (results not presented).

## Discussion

Using five waves of CHARLS data from 2011 to 2018 and hierarchical linear models, we analyzed how childhood maltreatment shapes the age trajectories of depressive symptoms in later life. We found that childhood intra- and extra-familial maltreatment are not only associated with higher depressive symptoms levels but also had an impact on the age trajectories of depressive symptoms. Specifically, peer bullying differences in depressive symptoms escalated with age for both genders. Physical abuse disparities in depressive symptoms remained stable with age in men, while they increase with age in women. Disparities in depressive symptoms associated with emotional neglect decline with age in men and initially increase then decrease in women.

Exposure to childhood maltreatment, both intrafamilial and extrafamilial, is associated with higher levels of depressive symptoms in later life. Regarding intrafamilial maltreatment, in the traditional Chinese culture, parental disciplinary actions such as corporal punishment were commonly accepted as a means of child-rearing [55], which can be aptly encapsulated by the proverb “Spare the rod, spoil the child”. Additionally, before the 1970s, families typically had multiple children [34] and parents had to work longer hours to support their families [40], which may make Chinese children of that era more vulnerable to parental emotional neglect. Previous research proposed that Chinese children tend to rationalize parental abusive behaviors and unconsciously defend their parents under the long-term influence of these cultural norms and social background [35]. Consequently, unlike the findings from Western developed countries such as Australia [32] and the Netherlands [20], it was expected that parental physical abuse and emotional neglect may not adversely affect later-life psychological well-being in China. However, our findings suggested that the enduring adverse effects of parental maltreatment on psychological well-being persist irrespective of the societal legalization of corporal punishment or the rationalizations for emotional neglect in the Chinese context. And importantly, results of this study highlight that the detrimental effects of parental maltreatment on mental health levels in later life do not vary from cultural contexts.

For extrafamilial peer bullying, we found that it was associated with higher depressive symptoms levels as well. Studies conducted in Western countries or meta-analyses have also come to the same conclusion [21, 26, 56, 57]. Furthermore, we identified that peer bullying has a more pronounced impact on late-life depressive symptoms than intrafamilial maltreatment, which is consistent with previous studies. For example, Tan and Mao [58] found that among the three types of maltreatment, bullying was the most prominent type in predicting depression, followed by parental physical abuse and emotional neglect. Using the CHARLS data, Shi and Yang [19] also found that peer bullying has a greater impact on depressive symptoms than parental physical abuse. One possibility is that bullying victimization may associated with further abuse by peers or adults, forming the first stage in a cycle of victimization that perpetuates itself over time and across situations [21]. Previous studies reported that over 40% of children who were bullied will continue to be bullied later in life [43, 59]. In addition, bullying victims are likely to become the “bully-victims” who also bully others, and both of them have been found to have elevated rates of adulthood depression [60]. This may be because childhood bullying victimization is often associated with a lack of social relationships, economic hardship, poor well-being, and negative stress response [11, 21, 60]. Those who were both victims and perpetrators tend to be frequently bullied by others and are the henchmen for the bullies, with few friends who would stand up for them [60].

Despite the detrimental effects of childhood maltreatment on average depressive symptoms levels being confirmed both in Chinese and Western contexts, it is still uncertain whether the levels of effects are consistent across countries. To date, China does not yet have effective measures to shield kids from bullying by their peers. Furthermore, before the implementation of the “One-child” Policy in the late 1970s and the Law of the People’s Republic of China on the Protection of Minors in 1991, Chinese children may have been more suspectable to parental physical abuse and emotional neglect. A meta-analysis suggested that people from Asian countries (mostly from China) experienced greater negative impacts of childhood maltreatment on self-esteem compared to their counterparts from North America [61]. Thus, comparative studies are needed to further examine differences between countries in the extent to which childhood maltreatment affects mental health in later life.

Expanding upon previous research, we found that childhood maltreatment also impacts the age trajectories of depressive symptoms in later life. Firstly, our results indicated that peer bullying differences in depressive symptoms escalate with age for both genders. We observed that disparities in depressive symptom levels associated with peer bullying increased by 87.50% in men (23.72% in women) from 45 to 60, and doubled in men (51.52% in women) from 45 to 75. Middle-aged and older adults may increasingly encounter negative events such as physical illness, bereavement, declined cognitive ability and unmet care needs as they age [54, 62]. Impairments in self-regulation and cognitive abilities resulting from adverse childhood experiences may lead to more adversities [25]. However, lower stress tolerance, negative coping strategies, and limited social support resources among bullying victims [19, 21, 23] may render them more susceptible to the influences of negative events, contributing to an acceleration in the growth rate of depressive symptoms compared to people without bullying experiences.

Furthermore, we found that physical abuse differences in depressive symptoms remained stable across ages in men and increased in women. This study revealed that during the 45–75 age range, differences in depressive symptoms levels between people with and without a history of physical abuse remained relatively constant among men, whereas the disparities grew nearly threefold among women. A Chinese study on chronic diseases came to a similar conclusion that differences in the number of chronic diseases between those who experienced physical abuse and those without widened with age [37]. In addition, prior research has identified that parental physical abuse during childhood can lead to faster deterioration of physical health in later life, such as physical functioning [36], multimorbidity [37, 38], and self-rated health [63]. Nevertheless, our robustness analyses indicated that health conditions cannot fully account for the association between childhood maltreatment and age trajectories of depressive symptoms. Further research is needed to explore the role of factors such as coping strategies and psychological resilience within this relationship.

However, the divergence trend in depressive symptoms related to physical abuse was limited to women in this study. A study from China also found that the divergence trend of multimorbidity associated with physical abuse is more pronounced in women than men [37]. Previous research has indicated that physical abuse experiences increase feelings of worthlessness and low self-esteem among victims, leading to negative self-evaluations and automatic thoughts [19, 64]. In China’s longstanding culture of “Son preference” that sons are more valued than daughters in a family [65], girls who have suffered from physical abuse may be more likely to internalize this negative experience as a reflection of their worthlessness and become activated by adverse events in later life. On the other hand, under the influence of “Son preference” culture, Chinese parents may use corporal punishment to motivate their sons to achieve greater achievements [65]. Thus, men may not attribute the physical abuse to their worthlessness but rather to disciplinary behaviors. This may contribute to gender differences in the age trajectories of depressive symptoms associated with physical abuse.

Lastly, emotional neglect disparities in depressive symptoms exhibited a gradual decline in men and an initial increase followed by a decrease in women with age. This study identified that within the 45–75 age range, differences in depressive symptoms levels associated with emotional neglect decreased persistently by approximately 60% among men, while among women, the disparities increased initially and then decreased, ultimately growing by nearly 2.5 times. Notably, the greater increase in depressive symptoms among people without emotional neglect experiences led to the decreased disparities in depressive symptoms among men. Additionally, we found that disparities in depressive symptoms linked to emotional neglect ultimately exhibited a convergence trend with age for both genders, which may explain why some previous studies did not detect a significant impact of emotional neglect on late-life depressive symptoms [33, 58]. This further underscores the necessity of investigating the impact of childhood maltreatment on age trajectories of depressive symptoms. However, the reduced disparities in depressive symptoms does not imply the obsolescence of intervention for emotional neglect. According to our results, emotional neglect continues to exert a significant impact on the average level of depressive symptoms in later life, particularly for women around 60. Furthermore, children born in recent cohorts will increasingly require more emotional attention from their parents since many Chinese families have only one child due to the “One-child” Policy.

Our findings on the age trajectories of depressive symptoms slightly differ from those in Western countries. For instance, Desch et al. found that for US adults aged 12–43, people who experienced physical abuse or neglect were more likely to exhibit depressive symptoms trajectories of “high but decreasing” and “increasing”, while those who had no such experiences were more likely to have trajectories of “consistently low” [66]. This suggests that differences in depressive symptoms levels associated with physical abuse and neglect may either increase or decrease with age. However, a study in the UK found that between the ages of 11 and 24, differences in depressive symptoms levels resulting from childhood trauma experiences initially increased and then decreased with age [67]. Additionally, a US study found that the difference in mean internalizing problems scores between those with and without a history of childhood maltreatment decreases with age in men but increases with age in women [68]. The reasons for the inconsistent results with ours may include: firstly, these studies only focused on depressive symptoms trajectories before the age of 45. Secondly, the measurement of childhood maltreatment was inconsistent among these studies and different from ours. Finally, these studies did not control for the cohort effects, a critical factor leading to inconsistencies in the health-age association [46, 47].

This study still has several limitations. Firstly, the information regarding childhood maltreatment in this study relies on retrospective recall by participants, which may introduce recall biases. Nonetheless, prior research has indicated that people tend to accurately remember their childhood experiences, making these retrospective reports reliable [69, 70]. Thus, recall bias does not appear to pose a significant threat to the validity and reliability of our results. Secondly, due to data constraints, we only utilized a single item to measure peer bullying and parental physical abuse, which may not accurately reflect the extent of childhood maltreatment, and did not investigate the effects of other forms of childhood maltreatment on depressive symptoms. Further research is needed to utilize more comprehensive scales to measure childhood maltreatment and examine the impacts of other maltreatment subtypes, such as emotional abuse, sexual abuse, and exploitation, on both average levels and age trajectories of late-life depressive symptoms. Lastly, we did not control for potential confounders in our main analyses. To ensure an adequate sample size in each age-cohort cell (*N* ≥ 30), we refer to previous studies [45, 71] and only focus on the variables of interest. In future research, it may be necessary to utilize datasets with larger sample sizes to examine the association while controlling for confounders.

Despite the limitations of this study, our findings advance our understanding of the impact of childhood intra- and extra-familial maltreatment on mental health trajectories and provide insights for tailoring life-course intervention programs for older adults’ mental health. Firstly, the government should establish the life-course intervention programs for mental health, mitigating risk factors such as childhood maltreatment at early life stages. Specifically, within the family context, the government should guide young parents in shifting away from traditional notions such as “Spare the rod, spoil the child” and “Son preference”; assist them in employing scientific parenting approaches and avoid inappropriate disciplinary actions such as corporal punishment; emphasize attentiveness and timely responsiveness to the physical and emotional needs of children, particularly for girls. Externally to the family, the government should establish peer bullying monitoring and prevention networks encompassing adolescents, families, schools, communities, and governmental agencies; enact legislation clearly defining bullying behaviors and holding perpetrators, guardians of perpetrators, schools, communities, or even local government accountable when bullying occurs. Secondly, for older people who have experienced childhood maltreatment, group interventions should be implemented to guide them to recollect both positive and negative memories from early life and integrate these memories into new life narratives, helping them develop psychological resources to reduce depressive symptoms and enhance well-being. Particularly, special attention should be given to bullying victims and women.

## Conclusions

The findings of this study indicate that childhood maltreatment is not only associated with poorer mental health in later life but also leads to increasing disparities in mental health as people age. It is urgent to conduct well-designed interventions targeting childhood maltreatment, which can not only protect children’s well-being but also effectively reduce the risk of late-life depression and improve the lifelong trajectories of mental health.

### Electronic supplementary material

Below is the link to the electronic supplementary material.

Supplementary Material 1

## Data Availability

The datasets supporting the conclusions of this article are available from https://charls.charlsdata.com/pages/data/111/zh-cn.html.

## Abbreviations

CHARLS: China Health and Retirement Longitudinal Study
CESD: Depressive symptoms
CES-D-10: 10-item Short Form of the Center for Epidemiologic Studies Depression Scale
HLM: Hierarchical Linear Models
MI: Multiple Imputation
IPW: Inverse Probability Weighting
SD: Standard Deviation
SE: Standard Errors
