# Supplementary material for: Childhood intra- and extra-familial maltreatment and later-life trajectories of depressive symptoms: evidence from China
Source: BMC Geriatr. 2024 Jul 12;24:598. doi: 10.1186/s12877-024-05169-w (PMC11241985; doi:10.1186/s12877-024-05169-w)
Supplement: Supplementary file 1 — Supplementary Material 1 [file 12877_2024_5169_MOESM1_ESM.docx]

**Table S1** Definition and measurement of variables used in the robustness checks

| Variables | Definition | Measurement |
| --- | --- | --- |
| Education | The highest level of education completed | 0 = elementary school and below;  1 = middle school and above |
| Smoking | Currently smoking or not | 0 = no; 1 = yes |
| Drinking | Currently drink any alcoholic beverages or not | 0 = no; 1 = yes |
| Social participation | Have you done any of these activities in the last month, including interacting with friends, playing cards, providing help, going to a sport club, joining a community-related organization, doing voluntary work, caring for a sick adult, attending an educational course, stock investment, using the Internet, and others? | The total score was calculated (0-9), with a higher score indicating higher levels of social participation |
| Self-reported health | How would you rate your health status? | 0 = very poor, poor, fair  1 = good, very good |
| Instrumental Activities of Daily Living | Do you have any difficulties with the activities, including doing household chores, preparing hot meals, shopping for groceries, managing assets, or taking medications? | The total score was calculated (0-20), with a higher score indicating higher levels of IADL limitations |
| Chronic diseases | Number of chronic diseases | 0-10 |

**Table S2** Results of the hierarchical linear model for age trajectories of CESD

| Variables | Men | | | | | | Women | | | | | |
| --- | --- | --- | --- | --- | --- | --- | --- | --- | --- | --- | --- | --- |
|  | M1 |  | M2 |  | M3 |  | M4 |  | M5 |  | M6 |  |
|  | β | SE | β | SE | β | SE | β | SE | β | SE | β | SE |
| Age | 0.015 | (0.022) | 0.025 | (0.025) | 0.005 | (0.022) | 0.029 | (0.025) | 0.053^*^ | (0.027) | 0.049^*^ | (0.025) |
| Cohort | -0.024 | (0.022) | -0.016 | (0.024) | -0.038^+^ | (0.021) | -0.025 | (0.023) | 0.007 | (0.025) | 0.000 | (0.023) |
| Age # Cohort | 0.002^*^ | (0.001) | 0.002^*^ | (0.001) | 0.003^***^ | (0.001) | 0.004^***^ | (0.001) | 0.003^***^ | (0.001) | 0.003^***^ | (0.001) |
| **Peer bullying** |  |  |  |  |  |  |  |  |  |  |  |  |
| Yes | 1.543 | (1.639) |  |  |  |  | -0.322 | (2.007) |  |  |  |  |
| Yes # Age ^a^ | 0.009 | (0.057) |  |  |  |  | 0.079 | (0.074) |  |  |  |  |
| Yes # Cohort | -0.017 | (0.053) |  |  |  |  | 0.053 | (0.064) |  |  |  |  |
| Yes # Age # Cohort | 0.002 | (0.002) |  |  |  |  | -0.000 | (0.003) |  |  |  |  |
| **Physical abuse** |  |  |  |  |  |  |  |  |  |  |  |  |
| Yes |  |  | 2.160^+^ | (1.274) |  |  |  |  | 3.150^*^ | (1.570) |  |  |
| Yes # Age |  |  | -0.034 | (0.044) |  |  |  |  | -0.048 | (0.056) |  |  |
| Yes # Cohort |  |  | -0.033 | (0.042) |  |  |  |  | -0.078 | (0.051) |  |  |
| Yes # Age # Cohort |  |  | 0.000 | (0.002) |  |  |  |  | 0.002 | (0.002) |  |  |
| **Emotional neglect** |  |  |  |  |  |  |  |  |  |  |  |  |
| Yes |  |  |  |  | -1.750 | (1.877) |  |  |  |  | 3.811^+^ | (2.035) |
| Yes # Age |  |  |  |  | 0.078 | (0.065) |  |  |  |  | -0.083 | (0.071) |
| Yes # Cohort |  |  |  |  | 0.106^+^ | (0.061) |  |  |  |  | -0.098 | (0.065) |
| Yes # Age # Cohort |  |  |  |  | -0.003 | (0.002) |  |  |  |  | 0.003 | (0.002) |
| Constant | 6.687^***^ | (0.654) | 6.209^***^ | (0.736) | 7.106^***^ | (0.639) | 8.206^***^ | (0.710) | 7.268^***^ | (0.758) | 7.550^***^ | (0.709) |
| Observations | 20690 |  | 20690 |  | 20690 |  | 22658 |  | 22658 |  | 22658 |  |

*Notes:* ^a^ # refers to the interaction term. CESD = Depressive symptoms. SE = Standard Errors. ^+^ *p* < 0.1, ^*^ *p* < 0.05, ^**^ *p* < 0.01, ^***^ *p* < 0.001

**Fig. S1** Predicted aging vectors of depressive symptoms: Control health conditions

*Notes:* Health conditions include self-reported health, Instrumental Activities of Daily Living, and number of chronic diseases. CESD = Depressive symptoms. Black lines = Exposed to childhood maltreatment. Gray lines = Not exposed to childhood maltreatment.
